# Diversity and Multigene Phylogeny of the Genus *Floccularia* (Agaricales, Basidiomycota)

**DOI:** 10.3390/jof11010074

**Published:** 2025-01-17

**Authors:** Zai-Wei Ge, Hua Qu, Malka Saba, Tian Gao, Martin Ryberg

**Affiliations:** 1 CAS Key Laboratory for Plant Diversity and Biogeography of East Asia, Kunming Institute of Botany, Chinese Academy of Sciences, Lanhei Road 132, Kunming 650201, China; quhua@mail.kib.ac.cn (H.Q.); gt2576975992@126.com (T.G.); 2 Yunnan Key Laboratory for Fungal Diversity and Green Development, Kunming Institute of Botany, Chinese Academy of Sciences, Kunming 650201, China; 3 University of Chinese Academy of Sciences, Beijing 100049, China; 4 Department of Plant Sciences, Quaid-i-Azam University, Islamabad 45320, Pakistan; rustflora@gmail.com; 5 Systematic Biology, Department of Organismal Biology, Uppsala University, Norbyvägen 18D, 75236 Uppsala, Sweden; martin.ryberg@ebc.uu.se

**Keywords:** Agaricaceae, Squamanitaceae, edible mushrooms, multi-locus phylogeny, new species, taxonomy

## Abstract

*Floccularia* is known as a northern-hemisphere-distributed genus with important economic values, especially in Western China. However, its species diversity in Asia and the phylogeny of this genus have not been critically studied. Based on worldwide sampling and multi-locus DNA sequence data (ITS, LSU, *rpb2*, *tef1*), the phylogeny of *Floccularia* was reconstructed, and the species diversity in Asia was critically studied on the basis of morphology and phylogeny. The results showed that five phylogenetic species can be recognized in this genus, of which there are four species in Asia, two species in North America and one species in Europe. According to our result, in addition to *F. luteovirens*, three new species, *F. asiatica*, *F. flava* and *F. sinensis*, were distributed in Asia, while in North America, *F. pitkinensis* and *F. fusca* could be synonyms of *F. albolanaripes*, as both species are phylogenetically intermingled within *F. albolanaripes*. Morphological descriptions of new species, color images of basidiomes, line drawings of their microscopic features, and a key to the Asian species of this genus are provided. Our study reconstructed the phylogeny of *Floccularia* for the first time and clarified the species diversity of *Floccularia* in Asia and suggests the need for detailed study of American specimens in order to accurately assess the diversity of *Floccularia* in America.

## 1. Introduction

The genus *Floccularia* Pouzar (Agaricales, Basidiomycota) was described by the Czech Mycologist Zdeněk Pouzar in 1957 to include *Armillaria straminea* P. Kumm., a synonym of *Floccularia luteovirens* (Alb. & Schwein.) Pouzar [1,2]. Members of *Floccularia* can be identified on the basis of the following characters: pileus medium to large, convex to applanate, covered with squamules; lamella adnate to adnexed, emarginatis; stipe central, with annulus formed by the very much developed veil; volva missing; spore print white to cream; basidiospores ellipsoid to broadly ovoid, smooth, without germ pore, amyloid; lamella trama parallel or almost parallel; squamules consist of short subcylindrical hyphae; clamp connections present; cystidia absent [3,4,5].

Species of *Floccularia* are widely distributed in the northern hemisphere, often growing alone or scattered in temperate to alpine terrestrial habitats [4]. Based on morphological features, *Floccularia* contains seven species. With ongoing study, *Floccularia subcaligata* (A.H. Sm. and P.M. Rea) Bon has been transferred to *Saproamanita* as *S. subcaligata* (A.H. Sm. and P.M. Rea) Redhead, Vizzini, Drehmel & Contu [6]; *Floccularia rickenii* (Bohus) Wasser ex Bon has been transferred to *Cercopemyces* as *C. rickenii* (Bohus) Dima & L. Nagy [7]; while a separate genus, *Leucopholiota* O.K. Mill., T.J. Volk & Bessette, was proposed to include *Floccularia decorosa* (Peck) Bon & Courtec. due to its special morphological characters [8]. Up to the present study, four species of *Floccularia* are recognized worldwide, *F. albolanaripes* (G.F. Atk.) Redhead, *F. fusca* (Mitchel and A.H. Sm.) Bon, *F. pitkinensis* (Mitchel and A.H. Sm.) Bon, and *F. luteovirens* [4,5,9].

Among the four recognized *Floccularia* species, *F. luteovirens* was originally described in Europe, and the remaining three species, *F. albolanaripes*, *F. fusca* and *F. pitkinensis*, were originally described in North America [9,10]. In Europe, *F. luteovirens* has been recorded in several countries, but it is very rare, and has been listed in the red list [5,11,12,13]. In North America, *Floccularia* species are limited to western regions ranging from the Rocky Mountains to the West Coast [9,10,14,15,16,17]. In Asia, *Floccularia* species are commonly recorded, with three species (*F. straminea*, *F. luteovirens* and *F. pitkinensis*) recorded in India [18] and two species (*F. luteovirens* and *F. albolanaripes*) in China [19].

As an edible species with both nutritional and medicinal properties, *Floccularia luteovirens*, known as “Huáng mó gū” (meaning “Yellow Mushroom”) in China, has been frequently collected in Western China by local people [20] (investigation by the first author), and its biology, ecology, and genetic variations have been studied [20,21,22,23]. Based on analyses of the nuc rDNA internal transcribed spacer region (ITS) and the nuc 28S rDNA (LSU), as well as two protein-coding genes (*rpb1* and *tef1*), considerable genotype diversity among *F. luteovirens* in the Qinghai-Tibet Plateau of Western China was detected [20,23,24]. However, the species diversity of *Floccularia* in China has not been critically studied, and only *F. luteovirens* and *F. albolanaripes* have been reported [19].

With the investigation of the species diversity of the Agaricaceae project going on, several specimens resembling *F. luteovirens* have been collected. A preliminary phylogenetic and morphological study revealed that a considerable degree of variation exists between Asian specimens and North American or European ones; further DNA sequences analyses have also exhibited significant differentiation, indicating the existence of more species in addition to *F. luteovirens*. Thus, the present study aimed to reconstruct the phylogeny of *Floccularia* based on a multi-locus (ITS + LSU + *tef1* + *rpb2*) data set and describe the new species based on morphological and phylogenetic evidence.

## 2. Materials and Methods

### 2.1. Morphological Studies

The specimens studied were deposited in the Herbarium of Cryptogams, Kunming Institute of Botany, Chinese Academy of Sciences (KUN, with HKAS numbers), the Denver Botanic Garden, Sam Mitchel Herbarium of Fungi (DBG), the University of British Columbia Herbarium (UBC), and the Uppsala University Herbarium (UPS).

Macromorphological characters of the basidiomes were based on field notes and images taken in the field. The color codes indicated were from Kornerup and Wanscher (1978) [25]. Microscopic characters were observed under a Leica DM2500 light microscope (Leica, Bensheim, Germany). Briefly, handmade sections of specimens were re-hydrated using 3% aqueous (*w*/*v*) potassium hydroxide (KOH), measurements were made after staining with 1% Congo Red reagent, and Melzer’s reagent was used to test the amyloidity of basidiospores. Basidiospores, basidia and pileal structures were measured, and line drawings were made.

For the basidiospore dimensions, the notation [n/m/p] indicates that the measurements were made for n basidiospores from m basidiomes and p collections. The notation (a)b–c(d) was adopted to represent the dimensions of basidiospores, with the range b–c indicating the minimum and maximum values of 90% of the value measured, and the a and d in parentheses indicating the extreme values; Q is used to indicate the length/width ratio of basidiospores in lateral view, while Qav represents the average Q of all basidiospores ± sample standard deviation.

### 2.2. DNA Extraction, PCR Amplifications and Sequencing

Total DNA was extracted from silica-dried tissue or herbarium specimens using a DNA extraction kit following the manufacturer’s instructions (BioTeke Corporation, Beijing, China). The internal transcribed spacer region (ITS), partial sequence of 28S rDNA (LSU), the most variable region of the second largest subunit of RNA polymerase II (*rpb2*), and partial sequence of the translation elongation factor 1-α (*tef1*) were amplified and sequenced. The primer pairs used for PCR and sequencing were ITS1F/ITS4 for ITS [26,27], LR0R/LR5 for LSU [28], bRPB2-6F/bRPB2-7.1R for *rpb2* [29], and EF1-983F/EF1-1567R for *tef1* [30]. For PCR amplification, the following thermocycler conditions were used: 5 min initial denaturation at 95 °C, followed by 34 cycles of 50 s at 94 °C, 40 s at 53 °C for ITS (50 °C for LSU, 58 °C for *rpb2*, 55 °C for *tef1*, respectively), 50 s at 72 °C, and a final extension of 8 min at 72 °C followed the last cycle. PCR products were purified and sent to Kunming Shuoqing Biotech Ltd. (Kunming, China) or Shanghai Sangon Biological Engineering Technology and Service Co. for sequencing. Sequence chromatograms were compiled with BioEdit v. 7.0.9 [31], and the newly generated sequences were deposited in GenBank (Table S1).

### 2.3. Phylogenetic Analysis

Two datasets, ITS dataset and the concatenated ITS-LSU-*tef1*-*rpb2* dataset, were compiled for the phylogenetic analyses to assist species identification and resolve their relationships. Initially, sequences of *Cystoderma aureum* (Bull.) Kühner and Romagn. and *Cystoderma amianthinum* (Scop.) Fayod were selected as the outgroups. Due to the hyper-variable sequences between the outgroups and the *Floccularia* species, the outgroups were removed in the final analyses, and a midpoint rooting strategy was adopted.

For the final ITS dataset, the sequences generated in this study were aligned with additional sequences downloaded from GenBank. Sequences were aligned using MAFFT v7.5.05 [32], and the alignments were manually adjusted to maximum alignment and minimize gaps by BioEdit v. 7.0.9 [31]. Phylogenetic analyses were performed using Bayesian inference (BI) and maximum likelihood (ML) methods, and clade received ≥70% ML Bootstrap (MLB) or ≥0.95 Bayesian posterior probabilities (BPP) was considered well supported. The best-fit substitution model for each alignment was estimated by PartitionFinder 2 [33]. Initial analyses showed that there were no hard conflicts among the four data sets; thus, the four gene fragments were concatenated using PhyloSuite v1.2.3 [34]. The resulting alignments and trees are available on Figshare at https://doi.org/10.6084/m9.figshare.27874062 (accessed on 14 January 2025).

ML bootstrap analysis was performed using GTRGAMMA as the best-fit likelihood model using RAxML 8.2.12 [35]. Statistical supports were assessed through 1000 rapid bootstrapping replicates. The BI analyses were performed with MrBayes v. 3.2.7 [36], applying GTR + G as the best-fit model. The initial 25% of sampled data were discarded as burn-in, and the Bayesian posterior probabilities (BPP) were calculated from the posterior distribution of the remaining trees.

## 3. Results

### 3.1. Phylogenetic Results

The aligned ITS dataset consisted of 103 sequences and 638 sites, of which 40 sites were parsimony-informative. The final matrix of the concatenated ITS-LSU-*tef1*-*rpb2* dataset included 39 ITS, 33 LSU, 34 *rpb2*, and 32 *tef1* sequences, with a total of 2717 characters (ITS 637 bp; LSU 872 bp; *rpb2* 717 bp; *tef1* 491 bp), of which 37, 7, 95, and 70 sites were parsimony informative for ITS, LSU, *rpb2*, and *tef1*, respectively.

**Figure 1:**
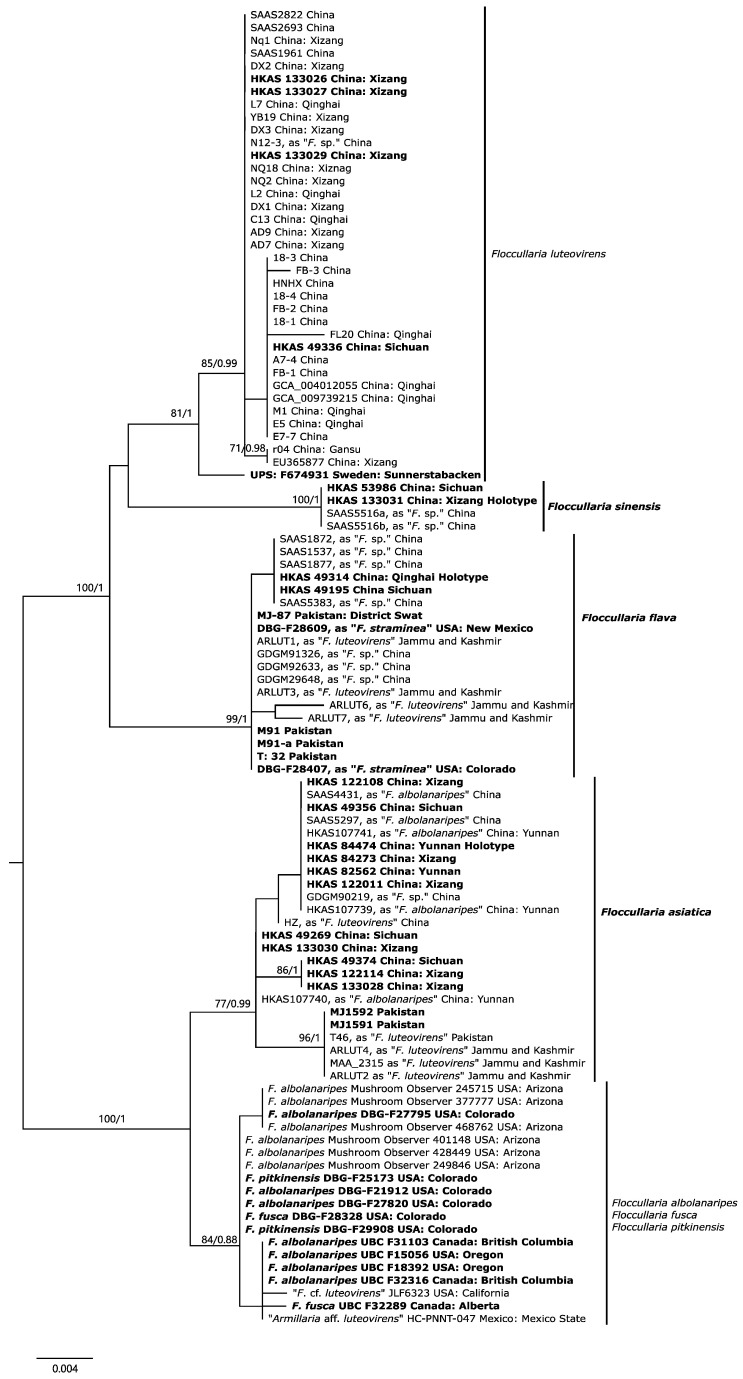
Best ML tree of *Floccularia* inferred from ITS data set. Node support is indicated beside branches. Newly generated sequences and new species proposed by this study are highlighted in bold.

Based on the ITS data set (Figure 1), which included the largest number of samples, the sequences that generated from Asian and European collections clustered into four well-supported clades: *F. asiatica* (MLB = 77%, BPP = 0.99), *F. luteovirens* (MLB = 81%, BPP = 1.00), *F. sinensis* (MLB = 100%, BPP = 1.00), and *F. flava* (MLB = 99%, BPP = 1.00). Meanwhile, the specimens from Western North America, identified as “*F. albolanaripes*”, “*F. fusca*” and “*F. pitkinesis*”, clustered within the same clade (MLB = 84%, BPP = 0.88) and intermixed (Figure 1).

**Figure 2:**
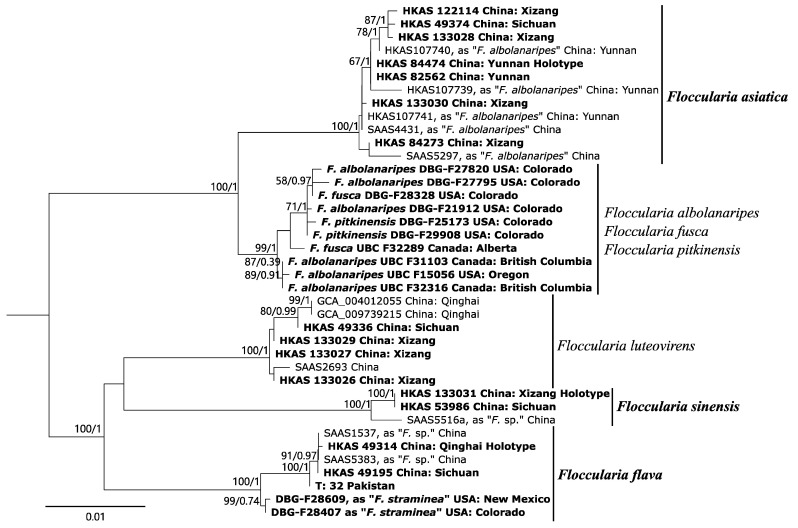
Best ML tree of *Floccularia* inferred from four concatenated loci (ITS, LSU, *rpb2*, and *tef1*). Node support is indicated beside branches. Newly obtained sequences and new species proposed in this study are highlighted in bold.

Similarly, based on phylogeny reconstructed from the concatenated ITS-LSU-*rpb2*-*tef1* data set (Figure 2), 39 sampled specimens representing five species formed five clades: the *F. asiatica* clade (MLB = 100%, BPP = 1.00), the *F. luteovirens* clade (MLB = 100%, BPP = 1.00), the *F. sinensis* clade (MLB = 100%, BPP = 1.00), and the *F. flava* clade (MLB = 100%, BPP = 1.00), while species from Western North America, *F. albolanaripes*, *F. fusca* and *F. pitkinensis*, clustered in the same clade and intermixed in a well-supported clade (MLB = 99%, BPP = 1.00). The relationships among the five clades are largely unclear. However, each of the five clades received strong statistical support (Figure 2). The *F. asiatica* clade contained samples from Asia (represented by samples from China, India and Pakistan). The *F. asiatica* is sister to *F. albolanaripes*, while *F. flava*, *F. sinensis* and *F. luteovirens* formed a well-supported clade, although the relationships among them are not clear.

### 3.2. Taxonomy

**Figure 3:**
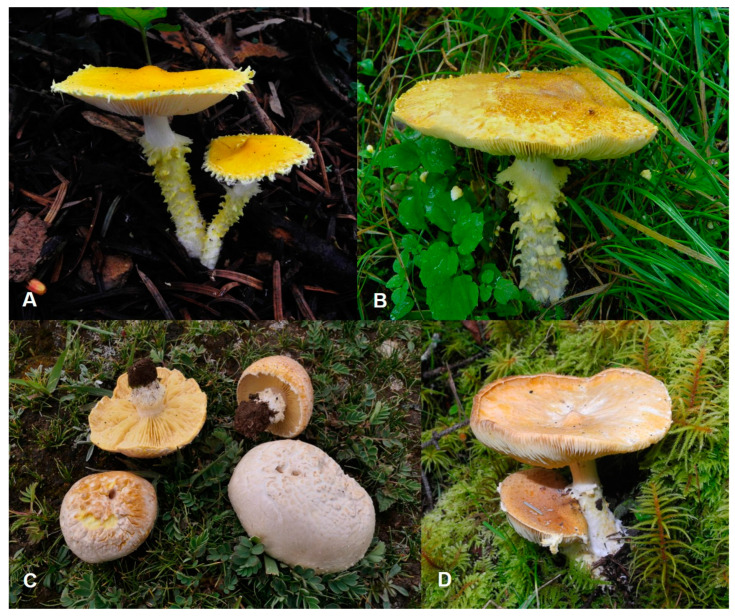
Fresh basidiomes of *Floccularia* spp. (**A**) *F. asiatica* (HKAS 84474, holotype). (**B**) *F. flava* (HKAS 49314, holotype). (**C**) *F. luteovirens* (HKAS 133026). (**D**) *F. sinensis* (HKAS 133031, holotype).

**Figure 4:**
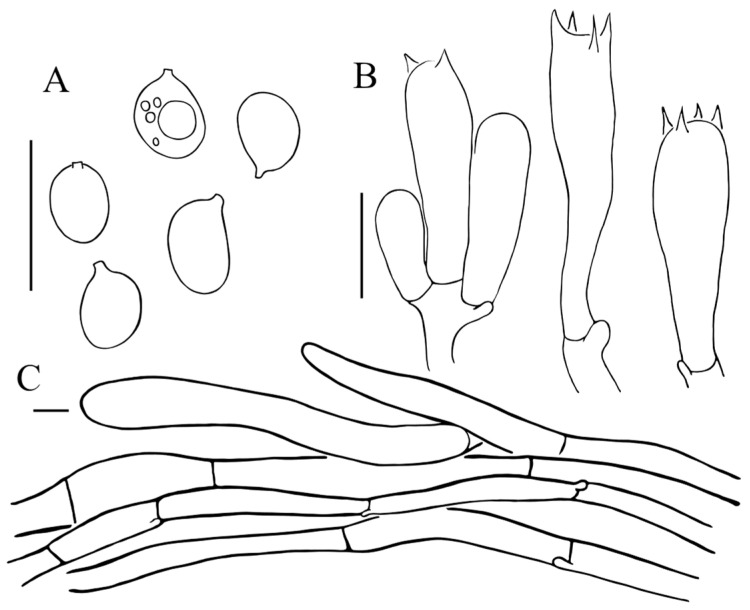
Micromorphology of *Floccularia asiatica* (HKAS 84474, holotype). (**A**) Basidiospores; (**B**) basidia; (**C**) terminal element of the squamules on pileus. Bars: 10 µm.

***Floccularia asiatica*** Z. W. Ge, Saba & H. Qu sp. nov. Figure 3A and Figure 4.

**Fungal Names:** FN 572231

**Etymology**: The specific epithet “*asiatica*” (Latin) refers to the continent from where it was collected.

**Diagnosis**: *Floccularia asiatica* differs from *Floccularia luteovirens* by its convex to planoconvex pileus, often with an umbo, prominent snow-white mycelium at the basal part of the stipe, Asian distribution, and preference for coniferous forests (sometimes intermixed with fagaceous trees). In addition, its ITS, LSU, *rpb2* and *tef1* sequences are obviously different.

**Type**: China, Yunnan Province, Dali Bai Autonomous Prefecture, Binchuan County, Jizu Mountain, E 100.389410, N 25.958555, in mosses in forest dominated by *Pinus yunnanensis* and *Quercus* sp., alt. 2290 m, 13 August 2014, Z. W. Ge 3636 (Holotype, HKAS 84474). GenBank: ITS = PQ640013, LSU = PQ639979, *rpb2* = PQ642192, *tef1* = PQ642224.

**Description**: Basidiomes slender. Pileus (3.5) 5–8 (10) cm, convex, planoconvex to applanate, at times reflexed in mature basidiomes, surface dry, pale yellow (3A3), light yellow (5D6), grayish yellow (2C5) to pastel yellow (2A4), or olive yellow (2C6), covered with light brown (5D6) to brownish orange (5C6–5C8), occasionally golden yellow (5B7) floccose squamules, with an obtuse umbo at disc, pileal margin appendiculate with white to yellowish white (4A1–4A2) veil remnants; Lamellae free to sinuate, crowded, white to yellowish white (1A2), margin eroded, up 0.4–0.8 cm in height, with 1–3 tiers of lamellulae. Stipe whitish to yellowish white (4A1–4A2), subcylindric, (4) 5–8 (10) × (0.4) 0.7–1.5 (2) cm, slightly thicker toward base, initially with a fragile and fugacious membranous veil between pileus margin and stipe; above annulus white, nearly glabrous, below annulus white, densely covered with reflexed, shaggy membranous, pale yellowish (4A2–4A3) to pastel green (30B4) squamules, surface of basal part obviously white, fibrillose to tomentose. Context fleshy, about 4 mm in the middle part of the pileus, white. Odor farinose, taste mild. Spore print white.

Basidiospores [59/2/2] (4.5) 5–6.5 (7) × (3) 3.5–4.5 µm, Q = (1.22) 1.25–1.75 (1.86), Qav = 1.48 ± 0.16, ellipsoid to broadly ellipsoid, colorless, thin-walled, amyloid, apiculus small. Basidia (18.5) 21–32.5 × 5.5–6.5 µm, clavate, hyaline, 4-spored, sterigmata 2–4 μm long. Lamellar trama parallel or almost parallel, made up of cylindrical colorless hyphae, 3.5–7.5 μm in diam. Cheilocystidia and pleurocystidia absent. Lamellar trama parallel or almost parallel, made up of cylindrical colorless hyphae, 3.5–7.5 μm in diam. Squamules composed of loosely arranged filamentous hyphae; sometimes slightly inflated, (5) 10–15 (20) µm in diam., smooth-walled, thin-walled to slightly thick-walled, colorless or sometimes with yellowish intracellular pigments. Clamp connections present in all tissues, abundant.

**Habitat and Distribution**: Found in forest dominated by *Pinus* sp. (sometimes intermixed with *Quercus* sp.) and *Picea* sp. in Asian countries. So far known in China, India and Pakistan.

**Additional specimens examined**: China, Sichuan Province, Ganzi (Garzê) Tibetan Autonomous Prefecture, Luhuo County, on the way from Luhuo to Wengda town [Seda (Sêrba) County], on soil in forest dominated by *Picea* sp., alt. 3410 m, 12 August 2005, Z. W. Ge 879 (HKAS 49374); Luhuo County, on the way from Wengda town [Seda (Sêrba) County] to Luhuo, in forest dominated by *Picea* sp., alt. 3775 m, 6 August 2005, Z. W. Ge 774 (HKAS 49269). Xizang Autonomous Region, Changdu (Chamdo) City, Leiwuqi (Riwoqê) County, Binda Town, in forest dominated by *Picea* sp. and *Betula* sp., alt. 3750 m, 16 August 2018, J. Wang 749 (HKAS 133028); same locality, X. H. Wang 5329 (HKAS 133030); Zuogong (Zogang) County, Zhayu Town, Xueba Village, in forest dominated by *Picea* sp., alt. 3780 m, R. Wu 107 (HKAS 122114), same locality, R. Wu 101 (HKAS 122108). Yunnan Province, Lijiang City, Yulong Naxi Autonomous County, Baisha town, Yufeng Temple, in forest dominated by *Pinus yunnanensis*, B. Feng 1456 (HKAS 82562). Pakistan: District Swat, 10 August 2018, Junaid Khan, MJ1591 (HKAS 145633).

**Notes**: *Floccularia asiatica* is similar to *F. luteovirens* in color and size of basidiomes. Both species generally form yellow flocculose-squamulose basidiomes. However, *F. asiatica* is often found in coniferous forests (sometimes intermixed with fagaceous trees), while *F. luteovirens*, originally described in Europe, inhabits mainly xerothermic grasslands and deciduous scrubs [1,5]. The pileus of *F. luteovirens* is hemispherical to plane (Figure 3C), and with pileus color fading to white with age, while those of *F. asiatica* are convex, planoconvex to applanate, with pileus color not fading with age, and often with an umbo at disc (Figure 3A); the basal part of the stipe of *F. asiatica* is prominently snow white (Figure 3A), fibrillose to tomentous, while that of *F. luteovirens* is mostly same as upper part of the stipe (Figure 3C). In addition, the ITS sequences of *F. asiatica* and *F. luteovirens* are quite different, with a difference of 26 nucleotides (606/632, 96%) between the holotype of *F. asiatica* and the *F. luteovirens* from the European collection.

*Floccularia asiatica* is also similar to the American species *F. albolanaripes*, *F. fusca*, and *F. pitkinensis* by their common yellow basidiomes. However, *F. albolanaripes*, described and distributed in Western United States, tends to become brownish to somewhat drab in maturity and often under conifer forests in the Rocky Mountains or under hardwoods (occasionally conifers) on the West Coast [9,10]. In addition, clamps present in *F. albolanaripes* are rare, while the clamp connections of *F. asiatica* are abundant in all tissues. *Floccularia asiatica* differs from *F. fusca* in the latter has entirely smoky gray basidiomes, a fuscous to cinereous pileus which is glabrous to inconspicuously squamulose, larger basidiospores measuring (5.5) 6–8 (9) × (3.7) 4–5 um, rare clamp connections, and a Western USA distribution [9].

**Figure 5:**
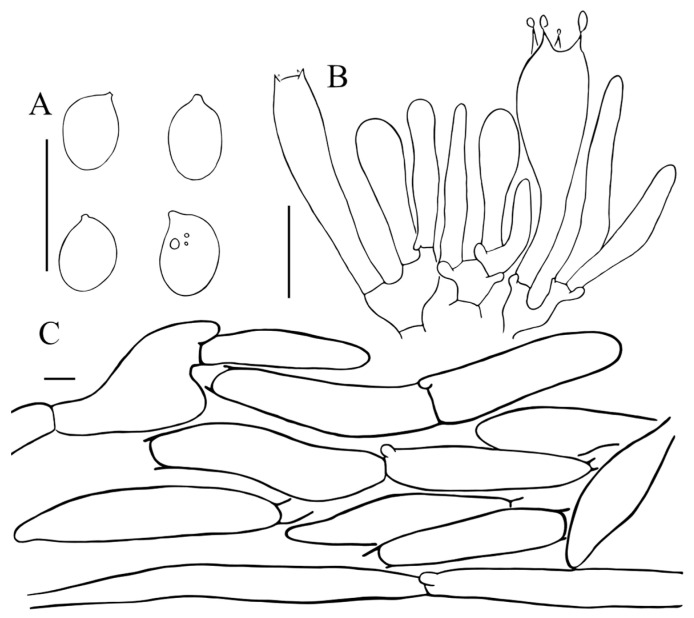
Micromorphology of *Floccularia flava* (HKAS 49314, holotype). (**A**) Basidiospores; (**B**) Basidia; (**C**) terminal element of the squamules on pileus. Bars: 10 µm.

***Floccularia flava*** Z. W. Ge & H. Qu sp. nov. Figure 3B and Figure 5.

Fungal Names: FN 572232

**Etymology**: *flava* (=Latin, adj.)–yellow, refers to the color of the basidiomes of this species.

**Diagnosis**: *Floccularia flava* differs from *F. asiatica* by the more conspicuous small pyramidal squamules, lower part of the stipe not obviously white, fibrillose to tomentous, and the pileus squamules composed of thicker hyphae. Its ITS, LSU, *rpb2* and *tef1* sequences are distinctively different from other species.

**Type**: China, Qinhai Province, Guoluo (Golog) Tibetan Autonomous Prefecture, Banma County, Nianlong Town, alt. 3780 m, in forest dominated by *Juniperus* sp., 9 August 2005, Z. W. Ge 819 (Holotype, HKAS 49314). GenBank: ITS = PQ640009, LSU = PQ639976, *rpb2* = PQ642189, *tef1* = PQ642214.

**Description**: Basidiomes slender. Pileus 6–11 cm in diam., initially plano-convex with an obtuse, expanding to applanate with age, sometimes shallowly depressed when mature; surface dry, yellowish white (3A2), greenish white (30A2), pale yellowish (1A3) to pale yellow (2A3), densely covered with more or less concentrically arranged, pyramidal squamules, color of the squamules range from grayish yellow (4C5), olive yellow (3C7), brownish orange (2A4), light brown (5C6) to golden brown (5D7), margin of the pileus with pastel yellow (2A4) to light green (30A5) veil remnants. Lamellae sinuate, crowded, up to 4 mm broad, yellowish white (1A2), to greenish white (29A2), with 1–3 tiers of small lamellulae, edge irregularly denticulate. Stipe central, subcylindrical, 5–8 × 1–2 cm, with a slightly bulbous base, white to whitish; annulus superior, membranous, ca. 1 cm away from the apex, cream to light yellow; covered up to the annulus with a dense coating of recurved floccose to shaggy, yellowish white to yellowish green (30A6), membranous squamules from the ruptured veil; basal part is not obviously white, fibrillose. Context whitish, fleshy. Odor fragrant, taste mild. Spore print white.

Basidiospores [62/3/3] (4.5) 5–6.5 (7) × (3) 3.5–4.5 (5) µm, 5.94 × 4.07, Q = (1.22) 1.29–1.71 (2), Qav = 1.47 ± 0.15, ellipsoid to broadly ellipsoid, hyalinous, thin-walled, amyloid, apiculus small. Basidia (22) 22.5–31.5 × (5) 5.5–9 µm, clavate, hyaline, 4-spored; sterigmata 2–5 μm long. Lamellar trama parallel or almost parallel, made up of cylindrical colorless hyphae, 4.5–12 μm in diam. Cheilocystidia and pleurocystidia absent. Pileus squamules composed of slightly inflated filamentous hyphae measuring 14.5–27 µm in diam., smooth-walled, slightly thick-walled, colorless. Clamp connections present in all tissues, common.

**Habitat and distribution**: Solitary or scattered on nutrient-rich soil in coniferous forest dominated by *Juniperus* sp., distributed in temperate zones of Asia and North America.

**Additional specimens examined**: China, Sichuan Province, Ganzi (Garzê) Tibetan Autonomous Prefecture, Shiqu County, Luoxu town, in forest dominated by *Picea* sp., alt. 3490 m, 30 July 2005, Z. W. Ge 700 (HKAS 49195). USA, New Mexico, Taos, Angustura Campground, Carson National Forest, DBG-F28609; USA, Colorado, Pitkin, City of Aspen, Main Street, DBG-F28407. Pakistan, Thandiani, 16 October 2021, Malka Saba/Alishba sheikh, T: 32 (HKAS 145632).

**Notes**: *Floccularia flava* is morphologically similar to *F. asiatica* in color and size of basidiomes. Both species share the yellowish pileus, lacking of cystidia and having clamp connections. However, *F. flava* can be distinguished by obvious small pyramidal squamules and a lower part of the stipe that is not obviously white fibrillose. In addition, pileus squamules of *F. flava* are composed of thicker hyphae measuring 14.5–27 µm in diam., compared to those of *F. asiatica* measuring (5) 10–15 (20) µm.

*Floccularia flava* is also morphologically similar to *F. albolanaripes* and sometimes specimens collected in Asia countries were mislabeled under this name. However, *F. albolanaripes*, originally described in Western North America, differs from *F. flava* by having hollow stipe, rare clamp connections in tissues, squamules consisted of narrower hyphae measuring 6–12 µm, and mostly associated with *Quercus* and *Abies* [9,10]. As shown in the phylogenetic tree, *F. albolanaripes* from Chinese collections distinctly belongs to *F. asiatica* species other than the American *F. albolanaripes* (Figure 1 and Figure 2).

**Figure 6:**
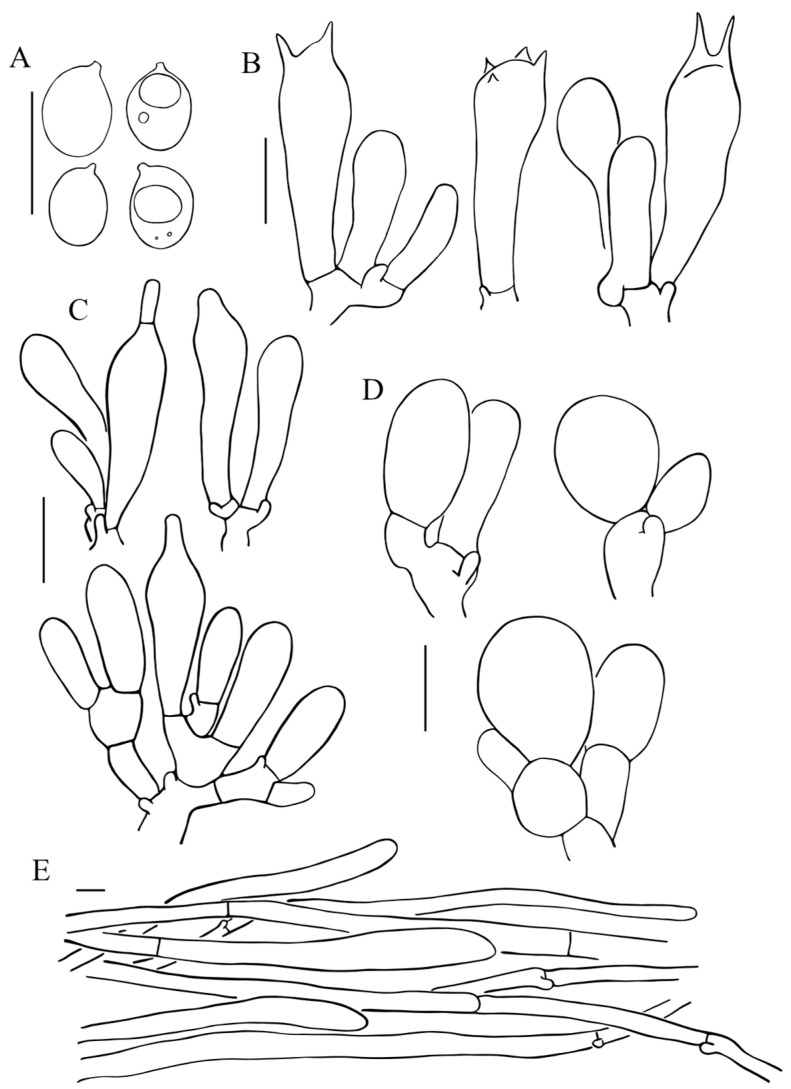
Micromorphology of *Floccularia sinensis*. (**A**) Basidiospores; (**B**) Basidia; (**C**) Pleurocystidia; (**D**) Cheilocystidia; (**E**) terminal element of the squamules on pileus. (**A**,**B**,**D**,**E**) from HKAS 133031 (holotype), (**C**) from HKAS 53986. Bars: 10 µm.

***Floccularia sinensis*** Z. W. Ge & H. Qu sp. nov. Figure 3D and Figure 6.

**Fungal Names:** FN 572233

**Etymology**: “*sinensis*” refers the locality where the type of this species was collected.

**Diagnosis**: *Floccularia sinensis* differs from other *Floccularia* species by having lageniform to narrowly clavate pleurocystidia often with rostrate to subcylindrical excrescence, the broadly clavate cheilocystidia, the 2-spored to 4-spored basidia, and the relatively bigger basidiospores. In addition, its ITS, LSU, *rpb2* and *tef1* sequences are obviously different from other species.

**Type**: China, Xizang Autonomous Region, Linzhi (Nyingchi) City, Chayu County, Chawalong Town, Mengzha Village, near Zhuangtong Bridge, E 98.19194, N 28.551839, alt. 3100 m, in mosses in forest dominated by *Tsuga* sp., 7 October 2019, Z. W. Ge 4755 (Holotype, HKAS 133031). GenBank: ITS = PQ640012, LSU = PQ639978, *rpb2* = PQ642191, *tef1* = PQ642218.

**Description**: Basidiomes slender, pileus 4.5–8 cm in diam, plano-convex, applanate to slightly reflexed when over mature, sometimes with an obtuse umbo at the disc; surface dry, orange white (5A2) to pinkish white (6A2), densely covered with appressed to slightly reflexed, light orange (5A4–6B4) to light brown 5D6–5D7) fibrillose squamules; margin appendiculate with loose patches of greenish white (30A2) to grayish green (30A3), membranous veil remnants. Lamellae sinuate, crowded, up to 3–6 mm broad, yellowish white (2A3), with 1–3 tiers of lamellulae, not discoloring on touch, with eroded edges. Stipe 4–4.5 × 0.5–0.7 cm, central, subcylindrical, broadened toward base (up to 1 cm), solid, upper half concolorous with pileus, lower half snow white; minutely flocculose above annular zone, with floccose-scaly annular zone, below annulus densely floccose, grayish green (30B4–30B6) to pastel yellow (1A3–1A4) squamules; basal part with distinct white mycelium. Context of pileus fleshy, cream, up to 3–6 mm thick. Odor indistinct; Taste mild. Spore print white.

Basidiospores [43/2/2] (5) 5.5–7.5 × (3.5) 4–5.5 (6) µm, 6.43 × 4.63, Q = (1.17) 1.25–1.63, Qav = 1.39 ± 0.14, ellipsoid to broadly ellipsoid, hyaline, smooth, thin-walled, amyloid. Basidia 24–32 (33) × 8–9.5 µm, clavate, hyaline, two spored, sometimes 4-spored; sterigmata 2.5–6 μm long. Cheilocystidia (12.5) 13–17.5 (19) × (6.5) 7–11.5 (13.5) µm, broadly clavate, hyaline, thin-walled; Pleurocystidia 19–24.5 (28) × 5.5–7 (8) µm, lageniform to narrowly clavate, with digitiform apex, (3) 3.5–6.5 (7) µm. Lamellar trama parallel or almost parallel, colorless, composed of cylindrical hyphae, thin-walled, 6.5–12 μm in diam. Squamules on pileus and stipe consist of loosely arranged, long subcylindrical hyphae, measuring 7–20.5 µm in diam., smooth-walled, slightly constricted at septa. Clamp connections present, abundant in all tissues.

**Habitat and distribution**: Solitary or scattered under conifers. Found in the southwest of China in August.

**Additional specimen examined**: China, Sichuan Province, Aba (Ngawa) Tibetan and Qiang Autonomous Prefecture, Maerkang (Barkam) County, Dangba Town, Yinlang Village, in mosses in forest dominated by *Pinus* sp., E 102.071944, N 31.731111, alt. 2500 m, 17 August 2007, Z. W. Ge 1901 (HKAS 53986).

**Notes**: The presence of cystidia and the 2-spored basidia are the most distinctive microscopic features of *F. sinensis*, which differs from all other *Floccularia* species as other species within this genus were recorded as having no cystidia [9,10]. Based on the multi-locus phylogeny (Figure 2), *F. sinensis* is closely related with *F. flava* and *F. luteovirens*, forming the sister to *F. luteovirens*, and these two species jointly forms the sister to *F. flava*. Macroscopically, *F. sinensis* is similar to these two species in having similar yellow to yellow brownish basidiomes. However, compared to *F. luteovirens*, basidiomes of *F. sinensis* is slender and its pileus does not fade to white with age (Figure 3); the squamules of *F. sinensis* is more or less appressed compared to the pyramidal squamules of *F. flava*. Most distinctively, *F. sinensis* can easily be recognized by having lageniform to narrowly clavate pleurocystidia which are often with rostrate to subcylindrical excrescence, the broadly clavate cheilocystidia (Figure 6), the 2-spored to 4-spored basidia, and the relatively bigger basidiospores measuring (5) 5.5–7.5 × (3.5) 4–5.5 (6) µm.

### 3.3. Key to Floccularia Species Distributed in Asia

1a. Basidiomes stocky, pileus hemispherical to applanate, fading to white with age, primarily in alpine meadows or near shrubs……………………*F. luteovirens*
1b. Basidiomes slender, pileus convex to broadly convex or nearly flat, pileus color not fading to white; primarily under Pinaceae (*Abies* spp., *Picea* spp., *Pinus* sp., *Tsuga* sp.), Fagaceae (*Quercus* sp.) or Cupressaceae (*Juniperus* sp.)…………………………………………………………………………………………………………2
2a. Presence of obvious cheilocystidia, basidia mainly two-spored…………………………………………………………………………………………………*F. sinensis*
2b. Without obvious cheilocystidia, basidia four-spored…………………………………………………………………………………………………………………………3
3a. pileus with an umbo, with appressed squamules of slender hyphae measuring (5) 10–15 (20) µm in diam.; surface of basal part of the stipe obviously white, fibrillose………………………………………………………………………………………………………………………………………………………………………*F. asiatica*
3b. pileus without umbo, with small pyramidal squamules of thicker hyphae measuring 14.5–27 µm in diam.; surface of basal part of the stipe covered with shaggy membranous, pale yellowish to pastel green squamules, not obviously white, fibrillose…………………………………………………………………………………………………………………………………………………………………………*F. flava*

## 4. Discussion

Species of *Floccularia* are commercially sold in local markets in China and Europe. The genus *Floccularia* can be recognized by its yellow-colored basidiomes. However, prior to the present study, species identification of this genus was mainly relied on morphological characters such as colors and the squamules of the pileus. The present study is the first phylogenetic study for this economically important genus. Our study, based on multiple gene loci analysis, identified five distinct clades within *Floccularia*, three species of which—the Asian–North American *F. flava*, the Asian *F. asiatica*, and the East Asian endemic *F. sinensis*—were described, significantly updating our understanding of *Floccularia* species diversity.

The newly described species, *F. flava*, is widespread in Asia and northern North America, extending from South Asia through East Asia to the West Coast of North America. Compared to the relatively wide distribution of *F. luteovirens* and *F. flava*, collections of *F. aisiatica* are restricted to Asia, and those of *F. sinensis* are entirely endemic to China, while collections of *F. albolanaripes*, *F. fusca* and *F. pitkinensis* are restricted to North America, suggesting that *Floccularia* species are geographically different.

The large number of *Floccularia* species and their diversity present in Asia suggest that Asia is a center of diversity for *Floccularia*. In addition to the widely distributed *F. luteovirens* and the three new species described in the present study, the existence of *F. pitkinensis* and *F. staminea* is also reported for Asia based on morphology [18]. However, the existence of *F. albonaltipes* in China has not been confirmed, as no Chinese specimens labeled as *F. albonaltipes* grouped together with the American *F. albonaltipes*. Interestingly, *F. sinensis* demonstrates the existence of cystidia, cheilocystidia and pleurocystidia; thus, the conception of *Floccularia* should be amended to include members with cystidia.

Three species are distinguished in North America based on morphological features: *F. albolanaripes*, *F. staminea* var. *ameriacana*, and *F. fusca* [9,10,15,17]. Morphological identification of *Floccularia* in the Americas is often difficult because microscopically defined characters are very much alike, and sometimes overlap, with no obvious differences [9,15]. Identification of *Floccularia* species mainly relies on macroscopic characters such as colors and the scaliness or fibrilloseness of the pileus surface. Indeed, Mitchel and Smith (1976) postulated that hybridization may occur in the central Rocky Mountain area, resulting in a “hybrid swarm” of *F. albolanaripes*, *F. staminea* var. *ameriacana*, and *F. fusca* [9]. In the present study, specimens identified as *F. pitkinesis* and *F. fusca* (Figure 1 and Figure 2) are phylogenetically intermingled within *F. albolanaripes*, forming a well-supported clade (Figure 2), suggesting that *F. pitkinesis* and *F. fusca* could be synonyms of *F. albolanaripes*. As these species were indeed intermingled in the same clade, a similar scenario has been recently shown in the phylogeny of the genus *Catathelasma* [37]. Whether these three species should be considered distinct species requires further sampling efforts and critical studies based on type specimens and materials collected at the type locality.

According to morphological studies [4,5,9,17], *Floccularia luteovirens* is considered to be a North Temperate species, and our present study confirmed its distribution in Eurasia based on ITS, LSU, *rpb2* and *tef1* sequences, while its distribution in North America has not been confirmed molecularly.

## Data Availability

The sequences presented in this study are openly available in https://www.ncbi.nlm.nih.gov/ (accessed on 14 January 2025) (see Table S1 for the accession numbers). The alignments and phylogenetic tree files are available in FigShare at https://doi.org/10.6084/m9.figshare.27874062 (accessed on 14 January 2025). All new taxa were registered in Fungal Names (https://nmdc.cn/fungalnames/) (accessed on 14 January 2025).

## Supplementary Materials

The following supporting information can be downloaded at: https://www.mdpi.com/article/10.3390/jof11010074/s1, Table S1: Voucher information and corresponding sequence data with GenBank accession numbers used in this study.
